# Changes in tibial cortical dimensions and density associated with long-term locking plate fixation in goats

**DOI:** 10.1186/s40634-023-00669-x

**Published:** 2023-11-07

**Authors:** Kristin M. Bowers, Lori D. Terrones, Xiaocun Sun, Rebecca Rifkin, Elizabeth Croy, Henry S. Adair, Pierre-Yves Mulon, Silke Hecht, David E. Anderson

**Affiliations:** 1 Large Animal Clinical Sciences, College of Veterinary Medicine, University of Tennessee, Knoxville, USA; 2 Office of Information Technology, University of Tennessee, Knoxville, USA; 3 Small Animal Clinical Sciences, College of Veterinary Medicine, University of Tennessee, Knoxville, USA

**Keywords:** Cortical porosis, Locking plate, Cortical density, Cortical bone loss

## Abstract

**Purpose:**

Cortical porosis, secondary to either vascular injury or stress-shielding, is a comorbidity of fracture fixation using compression bone plating. Locking plate constructs have unique mechanics of load transmission and lack of reliance on contact pressures for fixation stability, so secondary cortical porosis adjacent to the plate has not been widely investigated. Therefore, this study aimed to assess the effects of long-term locking plate fixation on cortical dimensions and density in a caprine tibial segmental ostectomy model.

**Methods:**

Data was acquired from a population of goats enrolled in ongoing orthopedic research which utilized locking plate fixation of 2 cm tibial diaphyseal segmental defects to evaluate bone healing over periods of 3, 6, 9, and 12 months. Quantitative data included tibial cortical width measurements and three-dimensionally reconstructed slab density measurements, both assessed using computed tomographic examinations performed at the time of plate removal. Additional surgical and demographic variables were analyzed for effect on cortical widths and density, and all cis-cortex measurements were compared to both the trans-cortex and to the contralateral limbs.

**Results:**

The tibial cis-cortex was significantly wider and more irregular than the trans-cortex at the same level. This width asymmetry differed in both magnitude and direction from the contralateral limb. The bone underlying the plate was significantly less dense than the trans-cortex, and this cortical density difference was significantly greater than that of the contralateral limb. These cortical changes were independent of both duration of fixation and degree of ostectomy bone healing.

**Conclusions:**

This study provides evidence that cortical bone loss consistent with cortical porosity is a comorbidity of locking plate fixation in a caprine tibial ostectomy model. Further research is necessary to identify risk factors for locking-plate-associated bone loss and to inform clinical decisions in cases necessitating long-term locking plate fixation.

**Supplementary Information:**

The online version contains supplementary material available at 10.1186/s40634-023-00669-x.

## Introduction

Internal fixation through plate osteosynthesis remains a mainstay for effective treatment of fractures, but fixation methods, plate composition and design, and surgical techniques have evolved over time [49]. Even in its earliest stages, orthopedic plate designs reflected the challenges at hand, each aiming to improve outcome and reduce complications. Landmark achievements include the development of metallic alloys for orthopedic plates, the establishment of the Arbeitgemeinschaft fur Osteosynthesefragen (AO) principles of effective fracture treatment, the invention of compression plates to encourage primary bone healing of fractures, the refinement of plate hole designs to allow for tension or dynamic compression plating, and the establishment of minimally invasive plate osteosynthesis [1, 40, 49]. Fixed angle devices, such as point-contact fixators and locking plates, were developed to respond to the challenges observed during conventional compression plating including marked soft tissue and periosteal damage during fixation, stress shielding of the underlying bone, and complications such as cortical porosis and/or refracture following plate removal [1, 12, 20, 29, 45].

Conventional compression plating aims to maximize fracture stability, minimize fragmentary motion, and minimize strain across the fracture gap to encourage primary bone healing, defined as the reestablishment of cortical bone without callus formation [7, 8, 22]. During early fixation, all load exerted on the bone is transferred from extremity to extremity through the plate and in compression plating, axial load is converted to shear stress [13, 27, 52]. Compression plates counteract these shear forces through frictional force generated by both plate-to-bone contact and summed screw torques, and once the external load exceeds frictional force, the strength of fixation directly reflects the axial stiffness of the screws furthest from the fracture site [1, 7]. Compression plating comorbidities and complications reflect these fixation biomechanics. Each screw is loaded individually at the screw-bone interface and repetitive interface loads can lead to screw toggle, failure, or pullout complications [1, 15]. These interfaces can be further compromised by cortical bone loss, either during the fracture itself or as a result of the fracture fixation (periosteal injury, soft tissue damage, or cortical stress-shielding) [1, 7, 20].

Cortical porosis is a comorbidity of compression plating [20]. Initially described as a cancellous transformation of cortical bone under the plate, cortical porosis represents a structural change to bone in contact with the plate characterized by bone resorption, medullary widening, and cortical thinning [31]. This comorbidity has been associated with increased refracture rates following plate removal and poses a challenge to clinicians who must balance radiographic fracture healing with increasing cortical porosity risk in their plate removal decisions [29, 48]. The definitive etiology of cortical porosis following compression plate fixation remains unclear [20]. Proposed etiologies include stress-shielding of the bone leading to disuse resorption or disruption of cortical perfusion either by surgical trauma or compression between the plate and periosteum [29, 49]. However, plate alterations aimed at reducing plate contact (e.g., limited contact dynamic compression plate) and/or stress differential between plate and bone (e.g., plate material compositions) have not succeeded in eliminating porosis and reducing refracture rates [3, 20, 21, 48].

In contrast to compression plates, locking plates do not depend on individual screw torques nor on plate-to-bone frictional forces for fixation stability [27, 47]. Locking plates act as single beam constructs in which there is no motion between the components of the beam (plate and screws), and axial external load is converted to compressive force, not shear forces [27, 28, 35]. The strength of locking plate fixation reflects the sum of all screw-bone interfaces, avoiding comorbidities of screw toggle and/or breakage and proving ideal for fracture cases with weak or lacking cortical bone (osteoporosis, comminution, etc.) [15, 28, 35]. Locking plates also are periosteal sparing, as the stability of locking plate fixation does not rely on frictional force generated by compressive contact between the plate and underlying bone [22, 29]. Generalized locking plate contact forces and surface areas have been described, but the true extent of locking plate contact is case-specific, depending on the fracture configuration, bone contours, cortical integrity, and overlying soft tissue [6, 12, 29, 51]. In theory, the differing contact profiles and fixation biomechanics between locking and compression plate fixation should reduce the incidence of cis-cortical porosis following locking plate fixation. However, Moens describes several clinical case series and studies that found no difference in cortical necrosis or porosity between locking and compressive plates [29]. Also, progressive bone atrophy (characterized by cortical thinning and porosis) following locking plate fixation of diaphyseal forearm fractures has been reported [18, 25, 32]. Moens challenged the claim that locking plates do not induce cortical porosis, citing a lack of in depth and rigorous comparisons between traditional compression and locking plates [29].

The current study aimed to assess the effects of long-term locking plate fixation in a caprine tibial segmental ostectomy model, focusing on cortical changes such as thinning, porosity, or loss of density under the plate as compared to both the trans cortex and to the cortical characteristics of the opposite limb. We hypothesized that long-term locking plate fixation would be associated with a loss of cortical integrity in the form of cortical thinning and decreasing cortical density of the bone underlying the plate. We hypothesized that these effects would be associated with time but would be independent of bone healing, fixation characteristics, and goat body weight.

## Materials and methods

### Goats

All study procedures were approved by the University of Tennessee Animal Care and Use Committee (protocol numbers 2741 and 2383). Boer-cross, adult goats (*n* = 160 females; mean weight 50.6 ± 7.58 kg, weight range 29 – 73 kg) were used in this study. Preoperatively, goats were housed in small group pens in groups of two to six (≥ 17 ft^2^ per goat); postoperatively, goats were housed individually in adjacent pens (≥ 20 ft^2^ per goat) for a minimum of seven days, followed by group housing based on goat behavior, clinical indication, and housing availability. Flooring included a layer of wood shavings laid on top of rubber mats over concrete flooring in a conditioned housing facility for the duration of the study. The goats were fed a balanced ration of grass hay, supplemental grain mix, and alfalfa as needed based on body condition. Free choice fresh water was provided via automatic waterers in group housing and in water buckets in individual pens. The goats used in this study were part of a series of orthopedic research projects assessing bone fillers in a tibial segmental defect model. Treatment groups included negative control (empty defect), positive control (autologous cortical bone graft), and intramedullary bone fillers with and without recombinant human bone morphogenic protein 2 (rhBMP-2). Goats from two projects in this series were included in this study, and cases were identified as either from Study A (2018) or Study B (2021); surgical technique, postoperative management, and postoperative monitoring did not differ between Studies A and B, but treatment group allocation and duration of the postoperative period differed by design. Criteria for inclusion of goats in this study included those having a segmental tibial ostectomy (2 cm defect, right hindlimb), bridging fixation using a single, custom designed locking plate, and computed tomographic examination of the operated limb immediately following plate removal. Exclusion criteria included goats diagnosed with surgical site infection, osteomyelitis, loose screws, broken screws, or cortical fractures during the study.

### Surgery

A mid-diaphyseal 2.0 cm segmental ostectomy was performed on the right hind tibia of each goat. The tibia was stabilized using a custom-designed low contact, round locking screw hole, double threaded 8-hole, 4.5-mm thick locking buttress plate with a solid portion between the 4^th^ and 5^th^ screw holes (Veterinary Orthopedic Implants, St. Augustine, FL, USA). Plate lengths varied based on tibia length, and plate selection (14 cm, 16 cm, or 18 cm) was made at the time of surgery following assessment of the tibia and surgical site. The plate was centered over the ostectomy and secured with eight 4.0-mm diameter self-tapping locking screws (Veterinary Orthopedic Implants, St. Augustine, FL, USA), four in the proximal tibial segment and four in the distal tibial segment. Each screw was tightened manually by the lead surgeon to ensure adequate engagement between screw and plate. Surgical procedures were performed as previously described [4]. Postoperatively, goats were fully weight-bearing and were maintained in full-limb bandages with medial and lateral plastic splints (Premier1Supplies, Washington, IA, USA) that spanned the limb from foot to femorotibial joint (knee). Bandages were discontinued between 2 and 4 weeks postoperative based on clinical condition as assessed by the supervising veterinarian. Goats were maintained in either group or individual housing as described above but no additional activity, behavior, or mobility restrictions were in place in the postoperative period. Goats were humanely euthanized at predetermined timepoints of 3, 6, 9, or 12 months postoperatively.

Radiographic examinations of the tibia, consisting of a minimum of two orthogonal views centered at mid-tibia, were performed immediately postoperatively and one day following surgery. Bone healing was assessed radiographically throughout the postoperative period at monthly or bi-monthly intervals as directed by the ongoing orthopedic research protocol. Computed tomographic examination of either the operated limb alone or both hindlimbs was performed at the time of plate removal, either postmortem or under general anesthesia as dictated by the experimental design. Computed tomography (CT) was performed using a 40-slice helical CT scanner (Philips® Brilliance-40™, Philips International B.V., Amsterdam, Netherlands). Prior to imaging, the CT scanner was calibrated according to manufacturer specifications. Transverse images were acquired as a multislice helical dataset and were reconstructed into 0.67-mm to 1-mm slice thickness using a bone algorithm. Images were uploaded into a picture archival and communications system (Sectra® PACS™ IDS7, Sectra AB, Linkoeping, Sweden) for further evaluation.

### Bone healing assessment

Per the ongoing orthopedic research protocols, each postoperative radiographic study and computed tomographic examination was reviewed by a board-certified veterinary radiologist (SH). Bone healing was assessed using an ostectomy gap filling score, referred to as “Bone Healing Score,” outlined in Table 1 and based on both the Radiographic Union Score for Tibial fractures (RUST) treated with intramedullary fixation and the modified RUST scoring system for plate fixation [10, 23, 50]. Scores ranged from 1 to 5 with a bone healing score of 5 representing complete filling of the ostectomy gap with bridging callus on all cortices. For both cortical thickness assessment and cortical density assessment using computed tomographic (CT) data, the bone healing score assigned to the respective CT examination was utilized for correlation analysis described below.

**Table 1:** Bone healing score values and criteria based on ostectomy gap filling

Ostectomy Gap Filling	Score
New bone filling < 25% of ostectomy gap	1
New bone filling 25–50% of ostectomy gap	2
New bone filling 51–75% of ostectomy gap	3
New bone filling > 75% of ostectomy gap but not completely healed	4
Ostectomy gap filled completely and/or bridging callus present on all cortices	5

### Cortical thickness assessment

**Fig. 1:**
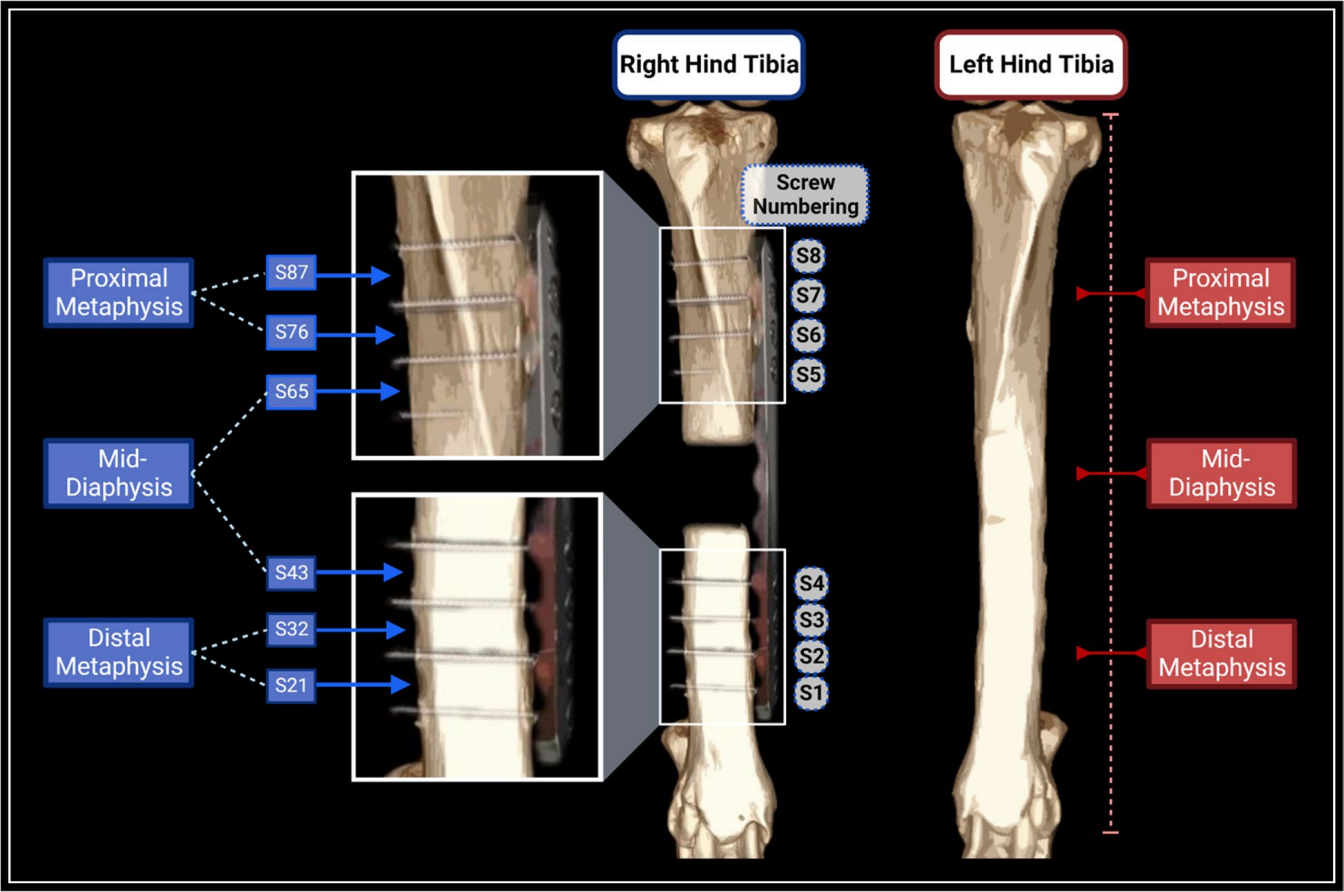
Illustration of measurement locations for operated and contralateral tibias. Contralateral limb measurement locations were determined using tibial length with each measurement point, indicated by a solid red line and label, representing a 25% increment in tibial length. Operated limb measurements were taken at the midpoint between each adjacent screw and grouped by anatomic localization (indicated by blue lines and labels). Screws were numbered for reference and each midpoint measurement location was identified using the two adjacent screws’ numbers. Figure created using BioRender

Cortical thickness and cortical density were measured using computed tomographic data of the operated and, if available, contralateral limbs obtained at the time of plate removal. Manipulation of image files, cortical thickness measurements, and cortical slab density measurements were performed using a picture archive and communication system (PACS) and digital radiographic imaging system (Sectra PACS, Sectra AB, Linköping, Sweden). Cis (medial cortex underlying the plate) and trans (lateral cortex opposing the plate) tibial cortical widths were measured using digital calipers on transverse CT frames (slice thickness 0.67–1 mm). For all operated limbs, standardized measurement loci were implemented using the visible screw tracts from locking plate fixation. Briefly, each fixation was performed using eight locking screws with bicortical engagement, four proximal to the ostectomy gap and four distal to the gap. The screws were referred to by number with screw 1 (S1) as the most distal screw and screw 8 (S8) as the most proximal screw (see Fig. 1). The midpoint frames between each adjacent pair of screws on the same bone segment were identified and utilized for measurements, yielding three measurement loci in the proximal tibial segment and three in the distal. For all contralateral limbs, three standardized measurement loci were determined using tibial length, measured in CT frames. Briefly, measurements were conducted at the appropriate frames representing ¼, ½, and ¾ tibial length to correspond to repeatable proximal metaphysis, mid-diaphysis, and distal metaphysis loci. On the contralateral limb, the medial cortical measurements were compared to operated cis-cortical measurements, and the lateral cortical measurements were compared to operated trans-cortical measurements.

At each measurement location, cis- and trans-cortical widths at the craniocaudal midpoint of the bone were recorded. Additionally, a qualitative binary assessment of cis-cortical irregularity was conducted by the reviewer with cortical irregularity defined as any disruption to the uniform tibial cortical structure such as bone porosity or periosteal new bone formation. Differences in cortical width were determined mathematically as trans width minus cis width.

### Cortical density assessment

Cortical density measurements were conducted at each operated and contralateral limb measurement location described above. To enhance density measurement accuracy and reduce frame-to-frame variability, density measurements were conducted as previously described on three-dimensional cylindrical cortical slab regions of interest (ROI) generated using Sectra PACS’s multiplanar reconstruction software (Sectra AB, Linköping, Sweden) [10, 14]. Slabs were generated using maximum intensity projections (MIP), slab thickness was set at 3 mm with 1 mm step, and ROI cylinders were generated with 2.5 mm diameter and 5 mm^2^ area. At each operated and contralateral limb measurement point, cis (medial) and trans (lateral) cortical slab densities at the craniocaudal midpoint of the bone were determined using manual placement of ROI cylinders within the appropriate cortex. Differences in cortical density were determined mathematically as trans density minus cis density.

### Statistical analysis

For both the operated and contralateral limbs, cis- and trans-cortical tibial cortical widths and densities were compared at each measurement locus using paired Student’s t tests. Additional demographic and surgical factors were screened for any effects on operated or contralateral tibial cortical width or density differences. These factors included body weight at study admission, orthopedic research project group (Study A or Study B), allocated treatment group, selected locking plate length, time in months that the locking plate was in place, cortical irregularity status, and bone healing score at the time of plate removal and CT exam. Descriptive statistics (including mean, median, standard deviation, frequency, percentile, and range) were generated as appropriate for the given dataset. The effects of cortical irregularity status, project group, and treatment group on width and density differences were analyzed using an analysis of variance (ANOVA), and the effects of numeric factors including body weight, plate length, bone healing score, and time were assessed using Pearson correlation analysis. For all demographic and surgical factors, effects were assessed first at the whole tibia level and then at each measurement locus individually. For ANOVA analyses, rank data transformation was applied when diagnostic analysis on residuals exhibited violation of normality and equal variance assumptions using Shapiro–Wilk test and Levene’s test. Post hoc multiple comparisons were performed with Tukey’s adjustment.

Operated and contralateral limb width and density differences were compared using paired Student’s t tests. Briefly, calculated width and density differences on the operated limb were matched with contralateral differences at the same location on the bone. The two proximal operated limb measurement loci were compared to the proximal metaphysis contralateral locus, the two loci immediately adjacent to the ostectomy site were compared to the contralateral mid-diaphysis locus, and the two distal operated limb measurement loci were compared to the contralateral distal metaphysis locus. Statistical significance was identified at *p* < 0.05. Analyses were conducted in SAS 9.4 TS1M7 (SAS institute Inc., Cary, NC) and IBM SPSS Statistics v. 28 (IBM Corp. Armonk, NY, USA).

## Results

### Goats

Out of a total population of 160 goats, 84 (mean body weight 50.11 ± 7.13 kg; range 35–66.6 kg) met the inclusion criteria for this study. Study groups, treatment groups, locking plate lengths, duration (in months) of plate fixation, and bone healing scores are presented in Table 2. Due to differences in experimental design between Study A (70 goats) and Study B (14 goats), significant differences in treatment group allocations and durations of fixation were observed (*p* < 0.001). Additionally, significant differences in allocated plate lengths and body weights at intake were noted between study groups (*p* < 0.001). Goats in Study A were slightly lighter than those in Study B (49.1 ± 6.6 kg and 55.4 ± 7.6 kg, respectively), and selected plate lengths were longer in Study A as compared to Study B (Supplemental Table 1).

**Table 2:** Descriptive statistics of demographic and surgical factors and results of correlation analysis with operated limb tibial cortical widths and densities. Statistical significance was identified at *p* < 0.05 and significant associations are denoted by bolded text

**Factors of Interest**			Operated Limb Cortical Width Difference Correlation	Operated Limb Cortical Density Difference Correlation
			*P-Value*	*P-Value*
**Orthopedic Study**			** < 0.001**	0.234
	*Number of Goats*	*Percent of Total*		
Study A	70	83.3		
Study B	14	16.7		
**Body Weight at Intake**			** < 0.001**	**0.048**
	*Mean* ± *St. Dev*	*Range*		
Body Weight (kg)	50.11 ± 7.13	35, 66.6		
**Locking Plate Length**			** < 0.001**	0.762
	*Number of Goats*	*Percent of Total*		
14 cm	13	15.5		
16 cm	21	25.0		
18 cm	50	59.5		
**Treatment Group**			0.457	**0.031**
	*Number of Goats*	*Percent of Total*		
Autologous Graft	5	6.0		
rhBMP-2 Bone Filler	30	35.7		
Bone Filler Alone	22	26.2		
Empty Defect	27	32.1		
**Time Locking Plate in Place**			0.310	0.591
	*Number of Goats*	*Percent of Total*		
3 months	14	16.7		
6 months	20	23.8		
9 months	24	28.6		
12 months	26	30.9		
**Bone Healing Score**			0.651	0.400
	*Number of Goats*	*Percent of Total*		
1	13	15.5		
2	3	3.6		
3	13	15.5		
4	19	22.6		
5	36	42.9		

### Tibial cortical width

**Fig. 2:**
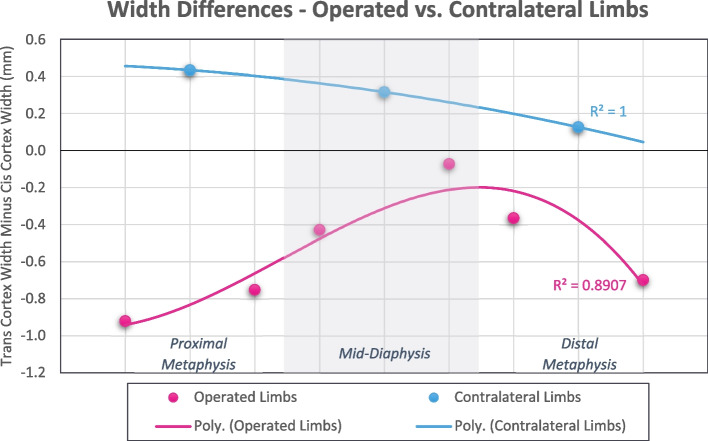
Comparison of cortical width differences between operated and contralateral tibias, organized by tibial regions. Polynomial lines of best fit were generated to aid in visualization and comparisons, and *R*^2^ values are included adjacent to the appropriate line

Operated limb cortical widths and width differences are presented for each measurement location in Table 3. At all points on the operated tibia, the cis cortex was significantly wider than the trans cortex (*p* < 0.001 for all comparisons). Mean contralateral limb cortical width differences, expressed as trans-cortex minus cis-cortex, are presented for each measurement locus in Table 3. At all locations on the contralateral tibia, the lateral cortex was significantly wider than the medial cortex (*p* < 0.001 for all comparisons). As described in Table 4 and illustrated in Fig. 2, mean cortical width differences were significantly different in magnitude and direction between operated and contralateral limbs (*p* < 0.001 for all comparisons), with the greatest disparity in cortical dimensions at the operated and contralateral proximal metaphyses (-0.836 ± 0.935 mm and 0.433 ± 0.412 mm, respectively). Qualitative cortical irregularity scores were significantly different between the operated cis-cortex and contralateral limb medial cortex (53.1% irregularity and 0.9% irregularity, respectively, *p* < 0.001).

**Table 3:** Operated limb mean tibial cortical widths and width differences, organized by location. Statistical significance was identified at *p* < 0.05 and significant differences between cis and trans cortical widths are denoted by bolded text

**Operated Limb Cortical Widths**					
**Tibial Regions**	**Measurement Points**	**Cis-Cortex Width (mm)**	**Trans-Cortex Width (mm)**	**Width Difference (mm)**	***P*-Value**
		*Mean* ± *St. Dev*	*Mean* ± *St. Dev*	*Mean* ± *St. Dev*	
Proximal Metaphysis	S87	4.842 ± 0.999	3.922 ± 0.727	-0.920 ± 1.019	** < 0.001**
	S76	4.871 ± 0.914	4.119 ± 0.659	-0.752 ± 0.846	** < 0.001**
Mid-Diaphysis Proximal to Defect	S65	4.818 ± 0.955	4.390 ± 0.633	-0.428 ± 1.049	** < 0.001**
Mid-Diaphysis Distal to Defect	S43	5.057 ± 0.658	4.986 ± 0.807	-0.071 ± 0.862	** < 0.001**
Distal Metaphysis	S32	4.962 ± 0.702	4.596 ± 0.668	-0.365 ± 0.685	** < 0.001**
	S21	5.144 ± 0.856	4.445 ± 0.589	-0.699 ± 0.860	** < 0.001**

**Table 4:** Comparison of cortical width differences between operated and contralateral tibias, organized by location. Statistical significance was identified at *p* < 0.05 and significant associations are denoted by bolded text

**Cortical Width Difference**			
	**Operated Limbs**	**Contralateral Limbs**	***P*-Value**
	*Mean (mm)* ± *St. Dev*	*Mean (mm)* ± *St. Dev*	
Proximal Metaphysis	-0.836 ± 0.935	0.433 ± 0.412	** < 0.001**
Mid-Diaphysis	-0.249 ± 0.970	0.317 ± 0.361	** < 0.001**
Distal Metaphysis	-0.532 ± 0.791	0.126 ± 0.236	** < 0.001**

On the contralateral tibia, the factors of treatment group, body weight, duration of locking plate fixation on the operated limb, locking plate length, and bone healing score of the operated limb were assessed for correlation with contralateral tibial width differences. No significant correlations were detected for treatment group, body weight, locking plate length, and time, but bone healing score on the operated limb was significantly correlated with contralateral limb mid-diaphyseal width difference (*p* = 0.042). As bone healing improved, the width difference at mid-diaphysis on the contralateral tibia decreased.

On the operated limb, the factors of orthopedic study group, body weight, locking plate length, treatment group, duration of locking plate fixation, and bone healing score were assessed for correlation with tibial width differences (Table 2). No significant correlations were detected for treatment group, time, and bone healing score. Study group, body weight, and locking plate length were each significantly correlated with operated tibial cortical width difference (*p* < 0.001 for each). The effects of study group were inconsistent when broken down by measurement foci. At the proximal metaphysis, goats from Study A had greater width differences than Study B, but at the mid-diaphyseal and distal metaphyseal loci, Study B had greater width differences. At the distal mid-diaphysis locus (S43) and distal metaphyseal locus (S32), increasing body weight was directly correlated in increasing width difference, but no significant effect of body weight was detected at the remaining measurement loci. Plate length was significantly correlated with width difference; as plate length increased, the operated tibial cortical width difference decreased.

Based on these results, further analysis of interactions between the demographic and surgical variables was performed. Bone healing score had no significant interactions with the other variables. Duration of locking plate fixation (time) was significantly associated with study group only (*p* < 0.001). The variables of study group, treatment group, body weight, and plate length were significantly codependent (*p* < 0.001 for all comparisons).

### Tibial cortical density

**Fig. 3:**
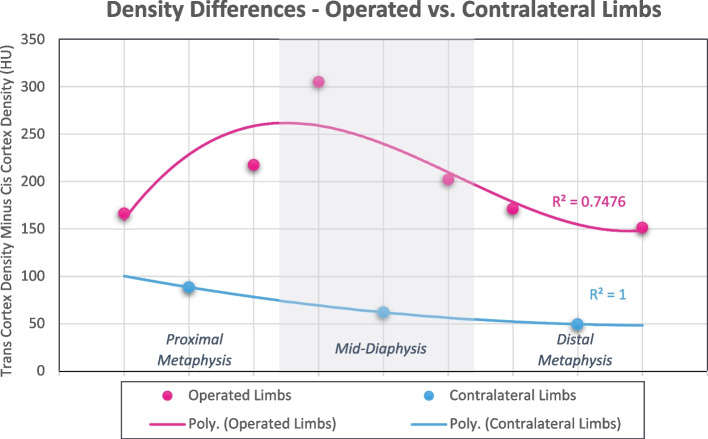
Comparison of cortical density differences between operated and contralateral tibias, organized by tibial regions. Polynomial lines of best fit were generated to aid in visualization and comparisons, and *R*^2^ values are included adjacent to the appropriate line

Operated limb cortical densities and density differences are presented in Hounsfield Units (HU) for each measurement location in Table 5. At all locations on the operated tibia, the cis cortex was significantly less dense than the trans cortex (*p* < 0.001 for all comparisons). Mean contralateral limb cortical density differences, expressed as lateral cortical density minus medial cortical density, are presented in Table 6. Similar to the operated tibia, the medial cortex was significantly less dense than the lateral cortex at all measurement points (*p* < 0.001 for all comparisons). As described in Table 6 and illustrated in Fig. 3, mean cortical density differences were significantly greater in the operated limbs compared to contralateral (*p* < 0.001 for all comparisons), with the greatest disparity in cortical densities at the mid-diaphyseal region.

**Table 5:** Operated limb mean tibial cortical densities and density differences, organized by location. Statistical significance was identified at *p* < 0.05 and significant differences between cis and trans cortical densities are denoted by bolded text

**Operated Limb Cortical Densities**					
**Tibial Regions**	**Measurement Points**	**Cis-Cortex Density (HU)**	**Trans-Cortex Density (HU)**	**Density Difference (HU)**	***P*-Value**
		*Mean* ± *St. Dev*	*Mean* ± *St. Dev*	*Mean* ± *St. Dev*	
Proximal Metaphysis	S87	1437.7 ± 226.3	1604.0 ± 232.4	166.2 ± 166.3	** < 0.001**
	S76	1471.8 ± 216.5	1689.3 ± 172.2	217.5 ± 125.4	** < 0.001**
Mid-Diaphysis Proximal to Defect	S65	1441.4 ± 278.3	1746.8 ± 134.7	305.4 ± 251.4	** < 0.001**
Mid-Diaphysis Distal to Defect	S43	1643.9 ± 154.5	1846.0 ± 127.4	202.1 ± 111.9	** < 0.001**
Distal Metaphysis	S32	1641.5 ± 143.9	1812.6 ± 124.9	171.0 ± 83.8	** < 0.001**
	S21	1638.2 ± 138.6	1789.3 ± 132.8	151.2 ± 92.7	** < 0.001**

**Table 6:** Comparison of cortical density differences between operated and contralateral tibias, organized by location. Statistical significance was identified at *p* < 0.05 and significant associations are denoted by bolded text

**Cortical Density Difference**			
	**Operated Limbs**	**Contralateral Limbs**	***P*-Value**
	*Mean (HU)* ± *St. Dev*	*Mean (HU)* ± *St. Dev*	
Proximal Metaphysis	192.0 ± 148.5	88.5 ± 48.2	** < 0.001**
Mid-Diaphysis	253.8 ± 200.2	62.2 ± 123.9	** < 0.001**
Distal Metaphysis	161.1 ± 88.4	49.5 ± 25.9	** < 0.001**

On the contralateral tibia, no significant correlations with cortical density differences were found for the factors of treatment group, locking plate length, and body weight. At the contralateral proximal metaphysis only, both bone healing score of the operated tibia and time that the locking plate was in place were significantly correlated with contralateral cortical density difference (*p* = 0.003 and *p* = 0.030, respectively). Greater bone healing scores, representing more complete bridging of the ostectomy, were associated with greater differences in density between the contralateral medial and lateral proximal metaphyseal cortices, but increasing duration of plate fixation was association with decreased density differences on the contralateral limb.

On the operated limb, the factors of bone healing score, locking plate length, study group, and time exhibited no significant correlation with cortical density differences (Table 2). Body weight at intake and assigned treatment groups were significantly correlated with whole-bone cortical density differences (*p* = 0.048 and *p* = 0.031, respectively). When broken down by measurement foci, increasing body weight at intake was significantly correlated with decreasing density differences at the mid-diaphysis proximal to the fracture gap (S65), but no additional significant interactions were noted. At two locations in the proximal metaphysis, significant differences in cortical densities were noted between the positive and negative control groups; no additional effects of treatment group reached significance.

## Discussion

Locking plate fixation combines the mechanical stability of an external fixator with the antimicrobial safety of an internal fixator [45]. Locking plate mechanics allow for stable fixation in cases of weak or limited cortical bone, and while they do not provide interfragmentary compression, locking plate fixations have similar strength, stiffness, and generalized success in terms of fracture healing and functional recovery when compared to matched compression plate fixations [2, 6, 33, 35, 46, 53]. Locking plate complications generally reflect those of all internal fixations, and the most common reasons for reoperation are infection, often associated with patient factors such as obesity, diabetes, smoking, or other underlying disease, and implant failure, most commonly associated with loss of screw purchase in compromised bone [30, 35, 36]. However, an increased rate of nonunion associated with locking plate fixation, particularly in distal femoral fractures (nonunion rates range from 14 to 41% of cases), has been identified, and upon further investigation, cases of cortical atrophy and loss of cortical integrity under the locking plates were observed, often without connection to the patient and infectious comorbidities described above [16, 25, 37, 38]. Also, cortical porosis and bone loss have been observed in cases of locking plate fixation of proximal humeral fractures, resulting in both nonunion and refracture complications [25, 32]. In a single-subject prospective trial, Hirashima et al. evaluated atrophic bone changes associated with locking plate fixation of paired radial and ulnar fractures without subsequent plate removal over 5 years of follow-up [18]. They reported a rapid loss of volumetric bone density in the radius and ulna under the plate (roughly 8% and 15%, respectively) during the first year of follow-up, relatively unchanged bone density between years 1 and 3, and rapid, marked declines in bone density from year 3 onward (roughly 34% volumetric bone loss under the plate in both the radius and ulna at year 5 of follow-up) [18]. Throughout this period, the volumetric bone mineral density outside of the margins of the plate did not significantly change [18]. Therefore, the goal of this study was to screen for cortical thinning, porosity, or loss of density following locking plate fixation of a caprine tibial defect.

Changes in cortical dimensions were assessed through measurements of operated and contralateral tibial cortical widths. Significant differences in cortical widths were noted at all measurement points on the contralateral tibia with the lateral cortex wider than the medial. These differences are expected due to normal tibial functional anatomy, tension surface, stress distribution, and muscular attachments, and similar relative cortical widths have been documented in focused caprine breed-associated anatomical studies [17, 24, 39]. However, the cortical geometry of the operated limb differed from that of the contralateral tibia both in magnitude and direction of cortical width differences (Fig. 2). Contrary to our hypothesis, the cis-cortex underlying the locking plates was significantly wider than the trans-cortex at all measurement points. Upon qualitative review, this wider bone was significantly more irregular and porous than the contralateral comparisons. Biomechanical analysis of tibial mechanics and strain distribution following plate removal was outside the scope of this study. Thus, we can only speculate from previous mechanical studies that the operated tibias would undergo markedly different patterns of tension, strain, and load distribution based on geometry alone, and the observed cortical irregularities may act as stress risers during load transfer as seen in previous osteoporosis models [9, 19, 34, 42, 43, 54]. Cortical widening in this study was rapid, and the duration of locking plate fixation had no significant effect on the geometric changes. In addition, operated limb cortical width differences were independent of bone healing score, suggesting that the fixation itself and not the degree of load sharing of the healed ostectomy was responsible for medial (cis) cortical widening.

Changes in cortical density and cortical porosity were assessed through multiplanar CT reconstruction and three-dimensional cortical slab density measurements. Similar to cortical widths, expected differences in cortical density between the contralateral tibia’s medial and lateral cortices reflect normal anatomic adaptations to the bone’s functions in load bearing and locomotion [10, 17]. In both the operated and contralateral limb, the cis-cortex was less dense than the trans-cortex, but the difference between three-dimensionally reconstructed cortices was significantly greater on the operated limb (Fig. 3). This translates to increased bone porosity and overall bone loss under the plate that corroborates what has been described following long-term locking plate fixation of human forearm and upper arm fractures [18, 25, 32]. Similar to cortical width differences, operated limb cortical density differences were not affected by either duration of fixation or bone healing score, suggesting rapid, fixation-associated changes that were independent of the defect’s degree of healing and load sharing status. We suggest that the significant correlations between operated cortical density differences and the variables of body weight at intake and treatment group are reflective of convenience sampling limitations discussed below. However, the interesting correlations between both cortical width and density differences and body weight is worthy of further investigation. In this study, as body weight increased, cortical width disparities between cis and trans at the mid-diaphysis increased while cortical density disparities at the proximal metaphysis decreased. Due to the codependence of body weight, study group, treatment group, and plate length in this study, further conclusions regarding the effect of body weight and, by extension, external axial load on locking-plate-associated cortical bone loss cannot be made, but this is a compelling topic for future research.

Another interesting finding worthy of further exploration is the significant correlation between bone healing score on the operated limb and cortical width and density differences on the contralateral limb. As operated limb healing scores improved, reflective of increasing load sharing of the defect, contralateral limb width differences decreased and density differences increased. Alterations in quadrupedal load distribution and biomechanical gait following induction of lameness have been well-described [11, 41, 44]. As the contralateral cortical measurements in this study were only intended to provide a contralateral comparison for observations in the operated limb, further exploration into lameness biomechanics and contralateral limb loads were outside the scope of this project. However, we hypothesize that these changes in contralateral tibial geometry and composition may reflect adaptive bone responses to alterations in loading environment as previously described [4, 11, 26].

Direct clinical comparison between the caprine model of bone healing utilized in this study and common clinical uses of locking plate fixation including distal femoral, radial, and humeral fracture stabilization is limited by differing biomechanical conditions. Indeed, the screw placement, plate location, and plate characteristics of distal femoral locking plates reflect the compressive loads exerted on the distal femurs as well as the cortical characteristics and soft tissue coverage of the human femoral metaphysis and epiphysis. In contrast, the screw placement, plate location, and plate characteristics of caprine tibial ostectomy fixation reflect the interplay of torsional, compressive, and shear forces exerted by the hindlimb mechanical axis and the characteristics of caprine diaphyseal bone. Thus, while cortical changes consistent with cortical porosis were observed in this model, further research is necessary to determine if these changes consistently occur in other weight-bearing and non-weight bearing environments as has been described in case reports and large-scale retrospective analyses [18, 25, 32, 37, 38].

The data from this study was obtained as a convenience sample from a series of orthopedic research projects using the caprine tibial ostectomy model. Thus, study population limitations including unequal distribution of study and treatment groups, lack of control for body weights and plate lengths, and overall codependence of study group, treatment group, body weights, and plate lengths are inherent. On individual factor analysis, orthopedic study group, body weight at intake, and locking plate length were significantly correlated with operated limb cortical width differences. However, due to the significant interaction effects among these variables and inconsistency of effect on cortical width differences when broken down by measurement location, conclusions regarding the influence of one variable over another and recommendations for future surgical planning cannot be made. To account for these inherent limitations, strict inclusion criteria were enacted to maximize uniformity in fixation mechanics, bone metabolism, and wound environment. This resulted in a relatively high exclusion rate (47.5%) and limitation of overall sample size, and we acknowledge these limitations to data interpretation. However, on post hoc sample size analysis with type II error set at 0.2, all demographic and surgical variable stratifications retained adequate power for whole tibial analysis and inclusion in our results. In addition, the original population of goats was utilized in a previous study by the same research group that assessed the correlation between cortical fracture incidence and surgeon-selected orthopedic plate length [5]. The exclusion criteria of the current study eliminated the fracture cohort from the previous work, limiting direct comparison between the effects of plate length in that analysis and the correlations between plate length and cortical widths in this analysis. Therefore, we recommend future prospective research to assess the effects of plate length and body weight on cortical dimensional changes associated with locking plate fixation.

In addition to the study population limitations discussed above, additional limitations in methodology and standardization must be acknowledged. This study compared measurements between the operated and contralateral limbs, but direct matching of measurement methods was not feasible due to the presence of screw tracts and gap ostectomies in the operated tibias. For that reason and to ensure standardized and repeatable measurements in both two- and three-dimensional reconstructions, the mathematical midpoints between screw tracts were used on the operated limb whereas relative tibial length was used on the contralateral tibias. Variations in plate lengths, plate positions, and tibial anatomy introduced minor and unavoidable variability in operated tibia measurements, and we acknowledge this as a limitation to the study. The duration of plate fixation was dictated by the ongoing research protocol, and we acknowledge that prolonged plate fixation of up to twelve months may limit direct clinical application of these results. All contralateral CT examinations were performed as a part of Study A, so no correlation analysis of study group effect was able to be performed on contralateral tibial measurements. Contralateral CT examination was not included in Study B’s methodology, and the limitations to contralateral dataset size and matching are acknowledged. Finally, due to the retrospective nature of this analysis on a convenience sample of goats enrolled in orthopedic research, no clinically normal hindlimb CT examinations from healthy, unoperated goats were available for inclusion in this analysis as unoperated controls, and we acknowledge this limitation to the interpretation of results.

## Conclusion

This study describes significant alterations in cortical dimensions and density following long-term locking plate fixation of caprine tibial segmental defects. Cortical porosis is an accepted comorbidity of compression plating, but due to locking plates’ unique mechanics and contact profile, it has not been widely investigated in association with locking plate fixation. In this study, the tibial cortex underlying the plate was significantly wider and more irregular than the opposite cortex at the same level, and this width asymmetry differed in both magnitude and direction from the contralateral limb. The bone underlying the plate was significantly less dense than the opposite cortex, and the magnitude of difference between operated cis and trans was significantly greater than that of the contralateral limb. This study provides evidence that cortical bone loss consistent with cortical porosity is a comorbidity of locking plate fixation and may contribute to nonunion or refracture complications in cases of extended duration of fixation. Further research is necessary to identify risk factors for locking-plate-associated bone loss and to inform clinical decisions in cases necessitating long-term locking plate fixation.

### Supplementary Information

**Additional file 1: Supplemental Table 1.** Plate length distribution between study groups. Selected plate lengths were significantly different between Study A and Study B (*p*<0.001).

## Data Availability

In accordance with institutional policy for patients’ medical records, data used in this study are not open to public but will be available upon motivated request to the corresponding author for the purpose of scientific research.

## Abbreviations

AO: Arbeitgemeinschaft fur Osteosynthesefragen
rhBMP-2: Recombinant human bone morphogenic protein 2
RUST: Radiographic Union for Tibial fractures
CT: Computed tomography
PACS: Picture archive and communication system
MIP: Maximum intensity projection
ANOVA: Analysis of variance
HU: Hounsfield Units
