# Supplementary material for: Changes in tibial cortical dimensions and density associated with long-term locking plate fixation in goats
Source: J Exp Orthop. 2023 Nov 7;10:111. doi: 10.1186/s40634-023-00669-x (PMC10634227; doi:10.1186/s40634-023-00669-x)
Supplement: Supplementary file 1 — Additional file 1: Supplemental Table 1. Plate length distribution between study groups. Selected plate lengths were significantly different between Study A and Study B (p<0.001). [file 40634_2023_669_MOESM1_ESM.docx]

**Supplemental Table 1:** Plate length distribution between study groups. Selected plate lengths were significantly different between Study A and Study B (*p*<0.001).

| **Study Group Plate Lengths** | | | | |
| --- | --- | --- | --- | --- |
|  | **14 cm Plates** | **16 cm Plates** | **18 cm Plates** | **Total** |
|  | *Number of Goats* | *Number of Goats* | *Number of Goats* | *Number of Goats* |
| Study A | 0 | 20 | 50 | **70** |
| Study B | 13 | 1 | 0 | **14** |
